# Routine activity effects of the Covid-19 pandemic on burglary in Detroit, March, 2020

**DOI:** 10.1186/s40163-020-00120-x

**Published:** 2020-06-23

**Authors:** Marcus Felson, Shanhe Jiang, Yanqing Xu

**Affiliations:** 1 Texas State University, San Marcos, USA; 2 Wayne State University, Detroit, USA; 3 University of Toledo, 2801 W. Bancroft St., Toledo, OH 43560 USA

**Keywords:** Routine activity, Disaster effects, Burglary rates, Urban crime distribution, Crime pattern theory

## Abstract

The spread of the coronavirus has led to containment policies in many places, with concomitant shifts in routine activities. Major declines in crime have been reported as a result. However, those declines depend on crime type and may differ by parts of a city and land uses. This paper examines burglary in Detroit, Michigan during the month of March, 2020, a period of considerable change in routine activities. We examine 879 block groups, separating those dominated by residential land use from those with more mixed land use. We divide the month into three periods: pre-containment, transition period, and post-containment. Burglaries increase in block groups with mixed land use, but not blocks dominated by residential land use. The impact of containment policies on burglary clarifies after taking land use into account.

## Introduction

The outbreak of the coronavirus pandemic began in China in late 2019 (Chen and Li 2020), then spread to the United States and other nations (Holshue et al. 2020). Although the Chinese government did not respond immediately, they soon imposed a massive lockdown policy to limit the spread of the virus (Fang et al. 2020). In response to this pandemic, a range of containment policies have emerged in many nations (Wilder-Smith and Freedman 2020). These policies led to quick transformations in routine activities, with many people staying at home, children out of school, and entertainment districts emptied. Non-essential businesses were closed, and many other businesses experienced substantial declines in the number of customers, while forced to deal with remaining customers on a reduced-contact basis.

Routine activity theory tells us that crime can be interpreted in terms of exposure to risk. Crime is most likely to occur when likely offenders converge in space and time with suitable crime targets in the absence of capable guardians against crime. For example, when youths are located near businesses whose owners and customers are absent, those businesses become excellent targets for crime. The Covid-19 epidemic shifted routine activities by moving owners and customers away from business locations, leaving them highly vulnerable to burglary and trespassing.

Soon after these routine activities changed, a variety of evidence began to emerge indicating dramatic decreases in crime (reviewed below). Supplementary evidence soon indicated that some types of crime were increasing in major ways, in contrast to the general reduction. These reports derived mostly from police data, as reported in news sources. However, news reports were supplemented by compilations and a few academic articles or drafts. The empirical task of the current research is to compare burglary changes in different zones of Detroit in response to the pandemic changes in routine activities. Before doing so, we review available evidence from other cities.

Indications of dramatic declines in crime in the United States emerged in news reports about New York (Jacobs and Barrett 2020), Los Angeles (Poston 2020) and St. Louis (Byers 2020). Academic work by Campadelli et al. (2020) adds to the evidence of a major overall crime drop in Los Angeles, especially for robbery, shoplifting, theft, and battery. However, they did not observe significant declines for stolen vehicles, burglaries, assault with a deadly weapon, or intimate partner violence. An academic compilation by McDonald and Balkin (2020) of crime in five major North American cities added evidence of major crime declines in Chicago, San Francisco and Philadelphia. Several specific index crimes declined notably in these cities. A multivariate analysis of crime changes in San Francisco (Shayegh and Malpede 2020) found a 43% decline in San Francisco neighborhoods. Declines were plotted day by day for the first 3 months of 2020, showing dramatic downturns in crime during the month of March, including thefts, weapons possession, homicide, domestic violence, traffic and sidewalk crimes, and (to a lesser degree) sex offenses. That study also found a 50% crime decline in Oakland, California.

The largest academic compilation on American cities comes from a British source. Ashby (2020) gathered data from eight large cities in the United States: Austin, Boston, Chicago, Los Angeles, Louisville, Memphis, Nashville, and Los Angeles. Full analysis was not complete for all of these cities. However, burglary data indicated declines for Austin, Los Angeles, Memphis, and San Francisco, but not for Boston or Louisville. Detroit crime statistics in the shadow of the pandemic have been limited, however a New York Times article (Eligon and MacFarquhar 2020) related that arrests from March 24 to April 14, 2020, were half as numerous as in the same period of 2019.

These news reports are not by any means limited to the United States. News reports from other developed nations also indicate major declines. Dodd (2020) reported major declines in the UK, while Dutch news reported a 46% drop in burglary, 43% decline in bike thefts, and a 74% drop in pickpocketing (Dutch News 2020). A project organized by Shayegh and Malpede (2020) is gathering daily crime reports in major European cities, however, these findings are not yet published. News reports from around the world confirm the pattern of general decline (see compiled Associated Press article by Danzio et al. (2020). An article in a South African newspaper, based on police data (Rall 2020), reports major declines in Carjacking (− 81%), non-residential robbery (− 66%), residential robbery (− 54%), rape (− 87%), major assault (− 85%), and several other violence categories.

Despite overall crime reductions in five cities, McDonald and Balkin (2020) found a hint of increased auto theft in Chicago. Byers (2020) found a major auto theft increase in St. Louis, contrary to the overall declines, a pattern also found in Ontario, Canada (Casey 2020). Other Canadian data fit the North American pattern, except that Toronto police found increases in speeding and stunt driving (Fox 2020). (The latter problems are also filtering in informally from police in the United States.) In another Canadian report, York Regional Police found overall crime declining by 13%, with traffic violations and impaired driving down by around 30% in apparent response to the Covid-19 situation. However, the same report indicated increases in domestic violence (Humphreys 2020). Similarly, data from Kansas City (Kizmar 2020) indicate major increases in domestic violence, a finding replicated around the United States (Bucchino 2020). These counter-trends justify further disaggregation of crime patterns and reductions in developed nations in response to changes in routine activities.

Perhaps the most interesting contrast from a routine activity perspective is the change in burglary rates. Total burglary rates did not change much, but when disaggregated by residential and commercial burglary, a different pattern emerged. The best evidence comes from commercial compilations by Pietrawska et al. (2020), who compared retail and restaurant victimizations to the larger city crime changes during the impact period. They find that retail and restaurant victimizations in Chicago declined quite dramatically during the impact period, with some notable exceptions. Robberies increased at locations that continued to operate, such as groceries and convenience stores. Although shoplifting declined greatly, vandalism increased. Most notably, retail burglary showed a strong upward trend even though overall burglary was declining in the larger city. This provide further reason to explore how the changes in routine activities have differential impact in different locations.

Shayegh and Malpede (2020) offer the most cogent evidence on this point—shifting crime hot spot maps before and after the Covid-19 shock in the cities of Oakland and San Francisco, California. The dramatic effect emerges for Oakland, which had a very strong crime concentration in its northwest section before “shelter in place” began. However, after this shift in routine activities, that hot spot disappears and spatial crime patterns, while not homogeneous, become much more evenly spread over urban space. This tells us to expect major changes in crime concentrations in some cities, these associated with land use differences in crime before and after the crisis period.

That finding raises an ancillary question: whether the dramatic shifts in routine activities resulting from the Covid-19 pandemic affect burglary rates differently in predominantly residential zones as opposed to zones with mixed residential and commercial land uses. We expect the shelter-in-place process to produce *more* burglaries in mixed land-use areas, where offenders can more easily reach commercial and industrial burglary targets. At the same time, we expect the shelter-in-place process to *decrease burglaries in residential zones*, where the increased guardianship during the Covid-19 crisis would lead to reduction in that crime. In other words, we expect the changes in routine activities engendered by the pandemic to produce burglary increases in some parts of a city even while burglary declines overall.

The focus on land uses and urban zones can readily be traced to crime pattern theory (Brantingham and Brantingham 1995, 1998, 1999). Although offenders often leave their zones of residence to find crime targets, they are also more likely to find those targets closer to home. Thus, the distance to crime is often moderate in length. In terms of the current research, areas with mixed land use provide more convenient burglary targets because commercial properties are less supervised by employees or customers and more readily subject to risk of burglary. At the same time, residential targets are more occupied and less available for attack. Offenders who live in zones with an appreciable number of commercial targets mixed among residences will have greater access to burglary targets. The changes in routine activities in light of the pandemic tell us to look for burglary increases mainly in urban areas not dominated by residences.

## Methods

The 2020 Covid-19 pandemic is a cruel natural experiment, testing how changes in routine activities alter crime rates. We have hypothesized that these activity changes are likely to have different effects in different parts of a city, depending on their land use.

### Covid-19 pandemic and regulations on routine activities in Detroit and Michigan

The first two confirmed Covid-19 cases were reported by the media on March 10, 2020 (WXYZ-TV 2020) although the first confirmed coronavirus case from the current state of Michigan official site appeared no later than March 1, 2020 (Michigan.gov 2020). One of the two cases came from Wayne county to which Detroit belongs. By the end of March, Michigan had 1179 confirmed Covid-19 cases, a majority of them from Metro Detroit (Michigan.gov 2020). Michigan then was one of the states having the highest confirmed cases.

On March 10, Michigan Governor Whitmer announced a state of emergency and closed all K-12 schools until April 6 (Fox17online 2020). On March 16, she made another announcement to partially close bars, restaurants, and entertainment venues for 2 weeks. Later she issued a series of executive orders to further ban events and gatherings. On March 24, Governor Whitmer issued a statewide stay-at-home order to ask residents to stay home unless they provide essential businesses (Mlive.com 2020). This order originally was effective for 3 weeks.

### Data and key measurement decisions

Our data were extracted from the Detroit Police Department crime incident report which was downloaded from the Detroit Open Data Portal. Detroit has 879 block groups. In this study we have focused on one aspect: whether an area is dominated by residential land use. We lack data that distinguish residential from commercial burglaries from one property to another. However, we do have data on how many parcels within a block group are residential or non-residential. Most of Detroit’s 879 block groups are more residential and less commercial. However, an appreciable number of these block groups are under 90% residential, allowing sufficient mixing of residences and business or industrial properties to enhance burglary opportunity during the crisis period. To simulate an experiment, we dichotomize block groups accordingly, comparing those with 90% or more residential properties to those with under 90%. When an area is at least 90% residential, that means there are at least nine homes for every business, making it more difficult to break into a business without at least one of the local residents noticing. When an area is less than 90% residential, that means that there are sufficient numbers of businesses for an offender to find a target with at least some near-by residents indisposed or distracted.

We have selected as our study period a single month during which dramatic changes in routine activities occurred—March, 2020. At the beginning of that month, remain-in-place orders were not issued. On March 10, those orders began to unfold, and on March 24 they were amplified more strongly by government and media sources. Therefore, we divide March into three periods: March 1 through 9, during which Detroit residents were only beginning to isolate; March 10 through 23, during which transition towards isolation was occurring; and March 24 through 31, during which isolation was most likely. This is a study of how crime changed during 31 days in March.

This research note offers a simple descriptive analysis. We only ask whether burglaries changed in two zones in Detroit during three periods in March, 2020. Given the short period, basic racial and social variables did not have time to change. Although economic conditions worsened for most of the population, those effects did not yet have time to play out during the short period under study. Many of the social problems in Detroit are chronic, having arrived well before Covid-19. The current paper focuses on the acute impact of the virus and the rapid shift in routine activities on burglary during 31 days in March, 2020.

### Analysis

Our research note is based on 360 burglaries reported to Detroit police in March, 2020. These burglaries were cross-tabulated by the three periods that reflect public and private response to Covid-19. Given the unequal numbers of days in the three periods, the burglaries per day were calculated for each. Table 1 gives the overall numbers, indicating 13 burglaries per day in Period A, 12 per day in Period B, and 9 per day in Period C. The rates per day per 100 block groups decline from 1.5 in Period A to 1.0 in Period C, approximately a 32% decline. However, the most interesting results emerge when separating residentially-dominated block groups from those with relatively mixed land use.

**Table 1:** Burglary risks during three periods in March, 2020, indicating the effects of stay-at-home practices responding to the Covid-19 pandemic, Detroit block groups

	Period A	Period B	Period C
	March 1 through March 9	March 10 through March 23	March 24 through March 31
a. Total block groups	879	879	879
b. Total days in period	9	14	8
c. Total burglaries in period	119	169	72
d. Burglaries per day (d = c/b)	13.22	12.07	9.00
e. Burglaries per day per 100 block groups e = (d × 100)/879	1.50	1.37	1.02
f. Per cent change from Period A	0	− 0.09	− 0.32

Table 2 presents the numbers for those 657 Detroit block groups with 90% or more residential parcels. Burglaries per day start as 1.57 in Period A, declining slightly in Period B. However, the end of March brings a 43% decline in the burglaries per day, less than one burglary per day per 100 block groups. In other words, removing the mixed land use block groups enhances to some extent the impact of Covid-19 on burglaries.

**Table 2:** Burglary risks during three periods in March, 2020, indicating the effects of stay-at-home practices responding to the Covid-19 pandemic, Detroit block groups with 90% or more residential parcels

	Period A	Period B	Period C
	March 1 through March 9	March 10 through March 23	March 24 through March 31
a. Total block groups in subset	657	657	657
b. Total days in period	9	14	8
c. Total burglaries in subset in period	93	131	47
d. Burglaries per day in subset d = c/b	10.33	9.36	5.88
e. Burglaries per day per 100 block groups in subset e = (d × 100)/657	1.57	1.42	0.89
f. Per cent change from period A	0	− 0.10	− 0.43

The most illustrative analysis emerges when we look only at the 222 block groups that have under 90% residential parcels. Unfortunately, the numbers are small, with only 89 burglaries to analyze. The main ones to observe are the 25 burglaries occurring during Period C. The burglaries per day rise during that period. The last row in Table 3 may be the most important, since it draws numbers from Table 2 as well. It calculates the ratio of each number in line (e) of Table 2 to its counterpart in line (e) of Table 3. Early in March, the burglaries per day per 100 block groups in predominantly residential areas totaled 1.57. The counterpart number for mixed areas was 1.3, producing a ratio of 0.83, reflecting the excess of burglaries in predominantly residential block groups. The ratio did not change appreciably in the middle period, but doubled in the end-of March period to 1.58.

**Table 3:** Burglary risks during three periods in March, 2020, indicating the effects of stay-at-home practices responding to the Covid-19 pandemic, Detroit block groups with under 90% residential parcels

	Period A	Period B	Period C
	March 1 through March 9	March 10 through March 23	March 24 through March 31
a. Total block groups in subset	222	222	222
b. Total days in period	9	14	8
c. Total burglaries in subset in period	26	38	25
d. Burglaries per day in subset d = c/b	2.89	2.71	3.13
e. Burglaries per day per 100 blocks in subset e = (d × 100)/222	1.30	1.22	1.41
f. Per cent change from Period A	0	− 0.06	+ 0.08
g. Ratio of burglaries in predominantly residential areas to mixed areas	3.58	3.45	1.88

That tells us that, in relative terms, burglaries have shifted away from predominantly residential areas to areas in which residences are substantially mixed with non-residential land uses. It is also important to compare row (d) in Table 2 to row (d) in Table 3. The comparison shows that burglaries per day decline in areas dominated by residential land use while, in the same time frame, burglaries per day rise in mixed areas at the end of March, 2020.^1^ We infer that a major shift in routine activities resulting from the Covid-19 pandemic influences the land use-crime relationship in Detroit. These findings are consistent with findings reviewed from other cities, showing commercial burglaries rising relative to overall burglaries as a result of changes in routine activities during the pandemic.

## Discussion

The changes in routine activities during the pandemic provides us with a natural experiment. Routine activities have changed exogenously; that is, a critic could not easily justify the argument that crime declines have impelled people to stay home. Nor can the declining crime rates be explained in terms of the usual social regularities. The racial composition of the population could not have changed appreciably within 31 days in March, 2020. Had we studied a full year or multiple years, such changes would be imaginable. However, the current period of study is quite brief.

The population was not undergoing reduced strain. The vast increase in job insecurity should have predicted increases in crime, not decreases. It makes no sense to argue that society’s basic social values—a long-term concept—were transformed in a few weeks. Nor can one readily explain why self-control would transform in such a short period.

We would argue that the main analysis problem in the current research stems not from control variables, but rather from the premature nature of the data reports and analyses. Not only can these data be revised later by police departments, but the coronavirus seriousness and public responses to it can themselves change. As a result, these conclusions can prove to be entirely premature. Our main purpose in writing a paper this early in the process is to set up a baseline for future analysis. Our results should be read in that context. At the time this paper was completed, Michigan’s restrictions on routine activities were loosened somewhat. However, the volatility of the routine activities confuses the natural experiment. We do not recommend extending the current analysis to April and May, 2020, until it becomes clarified how routine activities have changed in Detroit.

Some might interpret changes in terms of crime displacement. Given the net decline in burglary in Detroit during March, 2020, displacement is not evident as an overall conclusion. That is, the health crisis did not displace all or most of the burglary to new targets. However, we do not have data on which burglars were committing these offenses. It is possible that the same burglars were operating in all three periods, which is consistent with the conclusion that burglary was displaced, but at a lower rate than its initial level. It is also possible that a mix of old and new burglars were involved as March proceeded. In any case, the displacement argument cannot undermine the basic conclusion that, during this period in Detroit, burglary declined and shifted locations.

Some might argue that the collective threat produced greater social cohesion; indeed, many altruistic efforts are noteworthy during the pandemic. However, hording and fraud, along with counter trends in some types of crime—especially increases in domestic violence—hardly justify the conclusion that society is more cohesive overall. Nor does such cohesion explain why one type of burglary would go down while another type goes up. It is difficult to escape the conclusion that the early effects of the Covid-19 pandemic on crime largely rest on routine activity theory. It is also important to note that routine activity theory predicts where crime should go up and where it should go down and our findings are consistent with such predictions.

We cannot and do not claim that these results will hold for less developed nations, or for the future in developed nations. We do not insist that these results will hold as shutdowns of the economy drag on. The findings of the current paper cannot be generalized beyond Detroit without evidence from elsewhere. However, our research note offers some indicators that can be scaled up when additional data become available for more places and a wider span of time.

## Conclusions

The current research suggests that police departments and private security organizations should shift resources to businesses that are most vulnerable to attack, namely those relatively near to residential areas. The research also indicates that greater attention must be paid to shifting hourly and day of week patterns of commercial victimization to determine whether the pandemic has altered the temporal patterns along with locations of risk. If so, that would indicate that control strategies should change their temporal emphasis along with their spatial focus. We also advise close attention to those arrested to determine whether their journeys to crime sites have changed in response to the new opportunities and whether these changes might inform adult monitoring of youths.

## Data Availability

Data can be made available by the authors upon request.

## Footnotes

1 We also used 80% as a threshold to see the burglary pattern in the three periods of time. We found the same pattern. The total number of burglaries is smaller (with only 39 burglaries), but it is even more clear that burglaries per day per 100 block groups decline in areas dominated by residential land use for three periods of time (1.56, 1.39, 0.85) while burglaries per day per 100 block groups rise in mixed areas (0.94, 1.20, 2.71). Thus, this further examination suggests that the findings are not sensitive to the specific 90% cut-off used here.

## References

[CR1] Ashby MPJ (2020). Initial evidence on the relationship between the coronavirus pandemic and crime in the United States. Crime Science.

[CR2] Brantingham P, Brantingham P (1995). Criminality of place. European Journal on Criminal Policy and Research.

[CR3] Brantingham PL, Brantingham PJ (1998). Environmental criminology: From theory to urban planning practice. Studies on Crime and Crime Prevention.

[CR4] Brantingham PL, Brantingham PJ (1999). A theoretical model of crime hot spot generation. Studies on Crime and Crime Prevention.

[CR5] Bucchino, R. (2020). Domestic violence cases surge amid stay-at-home orders. The Hill. Retrieved from https://thehill.com/homenews/news/492506-domestic-violence-cases-surge-amid-stay-at-home-orders.

[CR6] Byers, C. (2020). When activity declines, so does crime: How the coronavirus pandemic is impacting crime in the St. Louis area. KSDK News website. Retrieved April 1, from https://www.ksdk.com/article/news/crime/coronavirus-impact-on-crime-st-louis/63-a2915ecf-8d37-4bab-9de5-0798984e685b.

[CR7] Campedelli, G. M., Aziani, A., & Favarin, S. (2020). Exploring the effect of 2019-nCoV containment policies on crime: The case of Los Angeles. Unpublished manuscript. Retrieved from https://arxiv.org/abs/2003.11021.

[CR8] Casey, L. (2020). COVID-19 has changed criminal behaviour and policing in Ontario. Canadian Press. October 32. Retrieved from https://toronto.ctvnews.ca/covid-19-has-changed-criminal-behaviour-and-policing-in-ontario-1.4880819.

[CR9] Chen Y, Li L (2020). SARS-CoV-2: Virus dynamics and host response. The Lancet Infectious Diseases.

[CR10] Danzio, S., Briceno, F., Tarm, M. (2020). Crime drops around the world as COVID-19 keeps people inside. Associated Press. Retrieved April 11, from https://www.sfgate.com/news/article/Crime-drops-around-the-world-as-COVID-19-keeps-15193839.php.

[CR11] Dodd, V. (2020). Crime in UK falls sharply since start of coronavirus lockdown. The Guardian. Retrieved from https://www.theguardian.com/uk-news/2020/apr/15/in-uk-falls-sharply-since-start-of-coronavirus-lockdown.

[CR12] Dutch News. (2020). 250 fined for breaking 1.5 metre rule, but ‘traditional’ crime drops sharply. Retrieved April 1, from https://www.dutchnews.nl/news/2020/04/250-fined-for-breaking-1-5-metre-rule-but-traditional-crime-drops-sharply/.

[CR13] Eligon, J., MacFarquhar, N. (2020). Coronavirus devastates Detroit police, from the chief on down. New York Times. Retrieved April 20. https://www.nytimes.com/2020/04/20/us/coronavirus-detroit-police.html?action=click&module=Top%20Stories&pgtype=Homepage.

[CR14] Fang, H., Wang, L., & Yang, Y. (2020). Human mobility restrictions and the spread of the novel coronavirus (2019-ncov) in China (No. w26906). National Bureau of Economic Research. Retrieved from https://www.nber.org/papers/w26906. 10.1016/j.jpubeco.2020.104272 PMC7833277 33518827

[CR15] Fox, C. (2020). Toronto police report more speeding, stunt driving during COVID-19 pandemic. CTV News. Retrieved from https://toronto.ctvnews.ca/toronto-police-report-more-speeding-stunt-driving-during-covid-19-pandemic-1.4879057.

[CR16] Fox17Online. (March 12, 2020). All Michigan K-12 schools to close through April 6. Retrieved from https://www.fox17online.com/news/national-news/coronavirus/all-michigan-k-12-schools-closed-through-april-6.

[CR17] Holshue ML, DeBolt C, Lindquist S, Lofy KH, Wiesman J, Bruce H (2020). First case of 2019 novel coronavirus in the United States. New England Journal of Medicine.

[CR18] Humphreys, A. (2020). Crime in a time of Covid-19: How the pandemic is changing criminality in our neighbourhoods. National Post, Canada. Retrieved from https://nationalpost.com/news/crime-in-a-time-of-covid-19-how-the-pandemic-is-changing-criminality-in-our-neighbourhoods.

[CR19] Jacobs, S. & Barrett, D. (2020). New York City’s crime rate plummets amid coronavirus shutdown. Washington Post, March 26. Retrieved from https://www.washingtonpost.com/world/national-security/coronavirus-new-york-city-crime/2020/03/26/6a408e94-6f9a-11ea-a3ec-70d7479d83f0_story.html.

[CR20] Kizmar, M. (2020). Domestic violence calls in KC have spiked during coronavirus lockdown. Kansas City Magazine. Retrieved from https://www.kansascitymag.com/domestic-violence-calls-in-kc-have-spiked-during-coronavirus-lockdown/.

[CR21] McDonald, J. F., & Balkin, S. (2020). The COVID-19 and the decline in crime. Unpublished paper. Retrieved from https://papers.ssrn.com/sol3/papers.cfm?abstract_id=3567500.

[CR22] Michigan.gov. (2020). Michigan data. Retrieved from https://www.michigan.gov/coronavirus/0,9753,7-406-98163_98173—,00.html.

[CR23] Mlive.com. (2020). Read Michigan Gov. Whitmer’s entire coronavirus stay-at-home order. Retrieved from https://www.mlive.com/public-interest/2020/03/read-michigan-gov-whitmers-entire-coronavirus-stay-at-home-order.html.

[CR25] Pietrawska, B., Aurand, S. K., & Palmer, W. (2020). Covid-19 and crime: CAP’s perspective on crime and loss in the age of Covid-19. Unpublished paper by request. Retrieved from https://capindex.com/.

[CR26] Poston, B. (2020). Arrests by LAPD and Sheriff’s Department drop amid coronavirus outbreak, Los Angeles Times. Retrieved from https://www.latimes.com/california/story/2020-03-18/lapd-arrests-crime-coronavirus.

[CR27] Rall, S. (2020). WATCH: Minister Bheki Cele notes a major decrease in crime since start of Covid-19 lockdown. The Mercury (South African News). Retrieved from https://www.iol.co.za/mercury/news/watch-minister-bheki-cele-notes-a-major-decrease-in-crime-since-start-of-covid-19-lockdown-47022987.

[CR28] Shayegh, S. & Malpede, M. (2020) Staying home saves lives, really! 10.2139/ssrn.3567394. PMC9428385 36061825

[CR29] Wilder-Smith A, Freedman DO (2020). Isolation, quarantine, social distancing and community containment: Pivotal role for old-style public health measures in the novel coronavirus (2019-nCoV) outbreak. Journal of Travel Medicine.

[CR30] WXYZ-TV. (March 10, 2020). First cases of coronavirus confirmed in Michigan. Retrieved from https://www.wxyz.com/news/coronavirus/first-cases-of-coronavirus-confirmed-in-michigan-one-each-in-oakland-and-wayne-counties.

